# Characterization of Tigecycline Resistance Among Tigecycline Non-susceptible *Klebsiella pneumoniae* Isolates From Humans, Food-Producing Animals, and *in vitro* Selection Assay

**DOI:** 10.3389/fmicb.2021.702006

**Published:** 2021-08-05

**Authors:** Mohaddeseh Moghimi, Mehri Haeili, Hanieh Mohajjel Shoja

**Affiliations:** ^1^Department of Animal Biology, Faculty of Natural Sciences, University of Tabriz, Tabriz, Iran; ^2^Department of Plant Biology, Faculty of Natural Sciences, University of Tabriz, Tabriz, Iran

**Keywords:** tigecycline resistance, *Klebsiella pneumoniae*, food animals, ramR, AcrR, AcrAB efflux pump

## Abstract

Emergence of extensively drug-resistant isolates of *Klebsiella pneumoniae* has prompted increased reliance on the last-resort antibiotics such as tigecycline (TGC) for treating infections caused by these pathogens. Consumption of human antibiotics in the food production industry has been found to contribute to the current antibiotic resistance crisis. In the current study, we aimed to investigate the mechanisms of TGC resistance among 18 TGC-non-susceptible (resistant or intermediate) *K. pneumoniae* (TGC-NSKP) isolates obtained from human (*n* = 5), food animals (*n* = 7), and *in vitro* selection experiment (*n* = 6). Isolates were genotyped by multilocus sequence typing (MLST). *ramR*, *acrR*, *rpsJ*, *tetA*, and *mgrB* (for colistin resistance) genes were sequenced. The presence of *tetX*, *tetX1*, and carbapenemase genes was examined by PCR. Susceptibility to different classes of antibiotics was evaluated by disc diffusion and broth macrodilution methods. The expression level of *acrB* was quantified by RT-qPCR assay. The 12 TGC-NSKP isolates [minimum inhibitory concentrations (MICs) = 4–32 mg/l] belonged to 10 distinct sequence types including ST37 (*n* = 2), ST11, ST15, ST45, ST1326 (animal isolates); ST147 (*n* = 2, human and animal isolates); and ST16, ST377, ST893, and ST2935 (human isolates). Co-resistance to TGC and colistin was identified among 57 and 40% of animal and human isolates, respectively. All human TGC-NSKP isolates carried carbapenemase genes (*bla*_OXA__–__48_, *bla*_NDM__–__1_, and *bla*_NDM__–__5_). *tetX*/*X1* genes were not detected in any isolates. About 83% of TGC-NSKP isolates (*n* = 15) carried *ramR* and/or *acrR* alterations including missense/nonsense mutations (A19V, L44Q, I141T, G180D, A28T, R114L, T119S, Y59stop, and Q122stop), insertions (positions +205 and +343), or deletions (position +205) for *ramR*, and R90G substitution or frameshift mutations for *acrR*. In one isolate *ramR* amplicon was not detected using all primers used in this study. Among seven colistin-resistant isolates, five harbored inactivated/mutated MgrB due to premature termination by nonsense mutations, insertion of IS elements, and frameshift mutations. All isolates revealed wild-type RpsJ and TetA (if present). Increased expression of *acrB* gene was detected among all resistant isolates, with the *in vitro* selected mutants showing the highest values. A combination of RamR and AcrR alterations was involved in TGC non-susceptibility in the majority of studied isolates.

## Introduction

Antibiotic resistance is rising to dangerously high levels in both human and veterinary medicine. The limited number of antibiotics coming to the market and new threats arising from extensively drug-resistant bacteria bring us perilously to the end of antibiotic era in which common treatable infections and minor injuries can once again be fatal. The use of human antibiotics in food animals as growth promoters or prophylactic agents has been identified as a significant contributing factor to increasing antimicrobial resistance (Van et al., 2020). Food animals have the potential to serve as reservoir for antibiotic-resistant bacteria, which can be transmitted to humans via direct contact or the food chain (Founou et al., 2016). *Klebsiella pneumoniae* is among the most problematic pathogens that have acquired much public health concern due to developing resistance to most clinically important antibiotics. It is an opportunistic pathogen that commonly causes a variety of the community- and hospital-acquired infections including urinary tract infection, pneumonia, and bloodstream infection. The emergence and dissemination of carbapenemase-producing strains of *K. pneumoniae* in healthcare facilities constitute a serious threat to public health (Pitout et al., 2015). The management of infections caused by carbapenem-resistant *K. pneumoniae* (CRKP) is complicated and often requires the use of last-resort antibiotics such as tigecycline (TGC) and colistin (Renteria et al., 2014; Sader et al., 2015). TGC is the first member of the novel class of glycylcyclines with expanded-spectrum antibacterial activity against Gram-negative and Gram-positive bacteria. It is approved by the Food and Drug Administration (FDA) for use in complicated skin and skin structure infections, complicated intra-abdominal infections, and community-acquired bacterial pneumonia (Beabout et al., 2015). TGC acts by inhibition of bacterial protein synthesis and has the ability to evade the classical mechanisms mediating resistance to tetracyclines including ribosomal protection and active efflux mediated by Tet proteins (Livermore, 2005).

In *K. pneumoniae*, TGC resistance is being increasingly reported since its approval (Spanu et al., 2012; Chiu et al., 2017a). TGC resistance in *K. pneumoniae* is believed to be mainly mediated by the overexpression of AcrAB efflux pump, which is regulated by local repressor AcrR and transcriptional activator RamA. The latter protein is also negatively regulated by RamR whose mutations have been found to contribute to significant increases in *ramA* and subsequently *acrAB* expression upon resistance occurrence (Hentschke et al., 2010; Chiu et al., 2017b). Decreased susceptibility to TGC has been also found to be related to alterations in the efflux pump encoding gene *tetA* (Du et al., 2018), or *rpsJ* (Beabout et al., 2015), the gene that encodes the ribosomal S10 protein. Moreover, enzymatic inactivation by TetX protein, a flavin-dependent monooxygenase, has been described to confer TGC resistance among some clinical pathogens (Moore et al., 2005; Deng et al., 2014). We aimed in the current study to investigate the TGC resistance determinants from a diverse group of TGC-non-susceptible (intermediate or resistant) *K. pneumoniae* (TGC-NSKP) isolates of clinical and animal origins and *in vitro* developed TGC-NSKP mutants. Also, the sequence types of TGC-NSKP isolates were determined by multilocus sequence typing (MLST) to identify major types associated with TGC non-susceptibility in each group of bacterial isolates from different host origins.

## Materials and Methods

### *Bacterial* Isolates

*Klebsiella pneumoniae* isolates from animal and human sources were included in this study. Animal isolates were obtained by taking cloacal swabs (using sterile cotton swab) from randomly selected broilers at a major chicken slaughterhouse. Taken samples were seeded on Eosin Methylene Blue (EMB) agar plates and were incubated at 37°C for 24 h. Screening for TGC-non-susceptible isolates among the grown colonies on EMB agar was performed using Mueller–Hinton broth (MHB) supplemented with 3 mg/l of TGC according to the method described in our previous study (Pishnian et al., 2019). Moreover, five clinical TGC-NSKP isolates obtained from patients hospitalized in two teaching hospitals of the country were included as human isolates. Identification of isolates was performed by conventional biochemical methods (Mahon et al., 2018).

### *In vitro* Selection of Tigecycline-Resistant Bacteria

To identify the genetic alterations mediating TGC resistance, upon TGC exposure, an *in vitro* resistance induction experiment was performed by exposing three TGC-susceptible (TGC-S) isolates to elevated concentrations of TGC. The Mueller–Hinton agar (MHA) plates supplemented with a sub-inhibitory concentration of antibiotic [1/2 × of minimum inhibitory concentrations (MICs)] were inoculated with 3 × 10^5^ CFU/ml TGC-S bacterial suspension and were incubated for 24–72 h. Colonies appearing on each plate were randomly picked and reisolated on media with the same concentration of TGC or concentrations, which were within 1.25–1.5× of previous concentration. In cases where no growth was observed at higher concentrations, colonies appeared at the same concentration were picked and transferred to MHA supplemented with the same and higher concentration of TGC. The obtained *in vitro* induced TGC-NSKP isolates were subjected to antimicrobial susceptibility testing.

### *Antimicrobial* Susceptibility Testing

The MICs of TGC (Glentham Life Sciences, Corsham, United Kingdom) (batch nos. 389SOI and 081GRB), colistin (colistin sulfate, Glentham Life Sciences, United Kingdom; batch no. 844WZQ), and imipenem (IPM) (Glentham Life Sciences, United Kingdom; batch no. 205WLC) were determined by broth macrodilution method using freshly prepared (less than 12-h-old) MHB from Difco (BD Diagnostic Systems, Sparks, MD, United States). The susceptibility to other classes of antibiotics was determined by disc diffusion method (Kirby–Bauer) according to the Clinical and Laboratory Standards Institute (CLSI) guidelines using the following antibiotics: gentamicin, amikacin, ampicillin, ceftriaxone, cefepime, nalidixic acid, ciprofloxacin, levofloxacin, gatifloxacin, tetracycline, doxycycline, minocycline, chloramphenicol, nitrofurantoin, and fosfomycin (BBL Sensi-Disc^TM^, Becton-Dickinson, Sparks, MD, United States). Due to the lack of established CLSI breakpoints for TGC at this time, the FDA breakpoints issued for Enterobacteriaceae (susceptible ≤ 2 mg/l, intermediate = 4 mg/l, and resistant ≥ 8 mg/l) were applied for interpretation of results. Isolates characterized with colistin MIC values greater than 2 mg/l were categorized as resistant according to guidelines described by the European Committee on Antimicrobial Susceptibility Testing (EUCAST). *Escherichia coli* ATCC 25922 was used as a quality-control strain for antimicrobial susceptibility testing.

### *Bacterial* G*enotyping by* Multilocus Sequence Typing

MLST with seven housekeeping genes (*rpoB*, *gapA*, *mdh*, *pgi*, *phoE*, *infB*, and *tonB*) was carried out for TGC-NSKP isolates of human and animal origins following the methods described previously (Diancourt et al., 2005). The allelic profiles and sequence types (STs) were assigned by using the *K. pneumoniae* MLST database provided by the Institut Pasteur, Paris, France.^1^

### *Molecular* D*eterminants of* Tigecycline, Col*istin, and* Carbapenem Resistance

The TGC-NSKP isolates were screened for the presence of *tetX* and *tetX1* genes by performing PCR with gene-specific primers (Table 1) [a second pair of primers were used for detection of *tetX1* gene (forward 5′-GCGACATTCCTGAACCAGAAACG and reverse 5′-CGGACGATTACTCTTCCAAGG)]. The coding regions of *ramR*, *acrR*, *rpsj*, *tetA*, and *mgrB* [for colistin (Col) resistance] were amplified and sequenced using the primers listed in Table 1. For isolates that did not yield a PCR product using primers targeting amplification of *ramR* coding sequence as well as some flanking regions (ramR-ext), amplification with other pairs of primers targeting an internal region of *ramR* coding sequence was repeated (ramR-int). Mutations were characterized by comparing the sequences with those of *K. pneumoniae* ATCC 700603 and three TGC-susceptible isolates (TGC MICs = 0.25 mg/l). The impact of the identified amino acid substitutions on the biological function of the protein (i.e., neutral or deleterious) were further predicted by using the Protein Variation Effect Analyzer tool (PROVEAN) (Choi and Chan, 2015). The IS Finder database^2^ was used to identify and analyze insertion sequences. Moreover, the presence of carbapenemase-encoding genes (*bla*_KPC_, *bla*_VIM_, *bla*_NDM_, and *bla*_OXA__–__48_) were examined by PCR using the primers and amplification conditions described previously (Poirel et al., 2011). The nucleotide sequences of *bla*_NDM_ were determined using the primers NDM-F-5′-GCCCAATATTATGCACCCGGTC and NDM-R-5′-AGCGCAGCTTGTCGGCCAT (Jafari et al., 2019).

**TABLE 1:** Nucleotide sequences of primers used in this study.

**Primer name**	**Sequence (5′–3′)**	**Size of product (bp)**	**References**
**Sequencing or detection**			
ramR-ext-F	TGGTCAGACGTGCCAAGATC	654	This study
ramR-ext-R	CAGTGTTTCCGGCGTCATTAG		
ramR-int-F	GCAAGCGTTACTGGAAGCTG	515	This study
ramR-int-R	CAAAGCCAAGGGCGATAATCT		
acrR-F	GTAAAGTCATTAACCTATGGCACG	667	This study
acrR-R	TTAAGCTGACAAGCTCTCCG		Rosenblum et al., 2011
rpsj-F	CAATCGTAATGGGTATGAGGAGT	514	This study
rpsj-R	CCTGAGTAACACGGTTTGCTT		
tetA-F	ACCCAACAGACCCCTGATCGT	1,133	This study
tetA-R	GCAAGTAGAGGGCAGCGCCT		
tetX-F	TTAGCCTTACCAATGGGTGT	243	Bartha et al., 2011
tetX-R	CAAATCTGCTGTTTCACTCG		
tetX1-F	TCAGGACAAGAAGCAATGAA	150	Bartha et al., 2011
tetX1-R	TATTTCGGGGTTGTCAAACT		
mgrB-F	ACCACCTCAAAGAGAAGGCGTT	347	Haeili et al., 2017
mgrB-R	GGCGTGATTTTGACACGAACAC		
**RT-qPCR**			
acrB-F	*C*AGCTTAACGCCTCGATCATC	127	This study
acrB-R	CCAGCTCAATTTTGGCGACATC		
rpsl-F	CCGTGGCGGTCGTGTTAAAGA	109	Cannatelli et al., 2013
rpsl-R	GCCGTACTTGGAGCGAGCCTG		

### *Assessment of acrB* Expression

To investigate the association between TGC non-susceptibility and overexpression of AcrAB efflux pump, the expression level of *acrB* gene was measured using RT-qPCR analysis. The total RNA from all TGC-S and TGC-NS bacterial cells was harvested using a GeneAll RiboEx Total RNA extraction kit (GeneAll Biotechnology, Seoul, South Korea). cDNA was synthesized from 1 μg of RNase-free DNase I (Takara Biotechnology, Dalian, China)-treated total RNA using Revert Aid first-strand cDNA synthesis kit (Thermo Fisher Scientific, Waltham, MA, United States). Real-time PCR amplification was performed using a Power SYBR green PCR master mix (Applied Biosystems, Foster City, CA, United States) on a Eco Real-Time PCR system (Illumina, San Diego, CA, United States). The relative gene expression levels were calculated using the 2^–ΔΔCT^ formula with *rpsL* housekeeping gene as internal control. A TGC-S isolate with TGC MIC of 0.25 mg/l was used as a reference strain. In the case of *in vitro* selected mutants, expression levels of *acrB* were compared with those of parental TGC-S isolates.

### *Nucleotide* Sequence Accession Numbers

The nucleotide sequences of the studied genes have been deposited at GenBank nucleotide sequence database under the following accession numbers:

MW653710 to MW653712 (mutated/altered *mgrB*), MW653713 and MW653714 (wild-type *mgrB*), MW653715 to MW653725 (mutated/altered *ramR*), MW653726 to MW653730 (wild-type *ramR*) MW653731 to MW653736 (mutated/altered *acrR*), MW653737 (HK10-S, AK88-S, AK298, AK299, and HK98), MW653738 (HK2-S), MW653739 (AK294 and AK297) (wild-type *acrR*), MW653740 (*rpsJ*, all isolates had identical sequences), and MW653741 (*tetA*, all isolates had identical sequences).

## Results

### *Bacterial* Isolates, Molecular Typi*ng, and* Antimicrobial Susceptibility Testing

Among the 1,430 samples taken from healthy broilers (collected from 83 different farms), six TGC-resistant (TGC-R) bacteria were detected, all corresponding to *K. pneumoniae*. The TGC-R *K. pneumoniae* (TGC-RKP) isolates displayed TGC MICs ranging from 8 to 32 mg/l. Moreover, one TGC-R isolate (AK513, TGC MIC = 8 mg/l) was included from our previous work (Pishnian et al., 2019), which was obtained from a turkey during the screening for colistin-resistant bacteria (Table 2). The seven TGC-R animal isolates belonged to six different sequence types including ST37 (*n* = 2 isolates) and ST11, ST15. ST45, ST147, and ST1326 (*n* = 1 each). Testing susceptibility to other antimicrobials revealed that TGC resistance was linked to a multidrug-resistant phenotype, and all animal TGC-RKP isolates showed resistance to quinolones, chloramphenicol, other members of tetracycline family and nitrofurantoin. The full resistance rate to gentamycin, ceftriaxone, and fosfomycin was found to be 42.8, 28, and 28%, respectively. One isolate, AK294, was found to be extended-spectrum β-lactamase (ESBL) producer using the combination disc method. Co-resistance to colistin and TGC was observed among 57% of animal isolates belonging to ST37, ST11, and ST15. All TGC-R animal isolates were susceptible to amikacin and IPM. Among the five studied human isolates, one was TGC-R (MIC = 8 mg/l), and the remaining isolates showed intermediate susceptibility to TGC (MICs = 4 mg/l). The MLST distributed the five TGC-NSKP human isolates into five distinct sequence types, including ST16, ST147 (also found in one animal isolate), ST377, ST893, and ST2935. Two human isolates showed simultaneous resistance to TGC (nonsusceptible), colistin, and IPM, with the remaining three isolates being characterized with co-resistance to IPM and TGC. Fosfomycin and amikacin were among the few antimicrobials that showed 40% activity against the TGC, IPM, and ±Col NSKP human isolates. The antimicrobial susceptibility profiles of the studied isolates are described in Table 2.

**TABLE 2:** Genotypic and phenotypic characteristics of tigecycline susceptible and non-susceptible *K. pneumoniae* isolates obtained from human, food animals, and *in vitro* selection assay.

**Isolate**	**Sequence type**	**MIC (mg/l)**			**RamR**	**AcrR**	**TetA**	**MgrB**	**Carbapenemase**	**Antimicrobial susceptibility profile**		
		**Tgc**	**Col**	**Ipm**						**S**	**I**	**R**
**Wild type isolates**												
AK88-S	ND	0.25	>128	0.25	WT	WT	–	WT	–	D, LVX, NA, TE, GM, MI, CRO, AN, GAT, F/M, FOS, C, CIP, FEP		AM
HK10-S	ND	0.25	0.25	0.25	WT	WT	–	WT	–	D, LVX, NA, TE, GM, MI, CRO, AN, GAT, F/M, FOS, C, CIP,FEP		AM
HK2-S	ND	0.25	0.25	1	WT	WT	–	WT	–	AN, FOS, TE, D, MI		AM, CRO, F/M, NA, FEP, GM, CIP, LVX, GAT, C
**Laboratory induced mutants**												
AK88-R1	ND	16	>128	0.25	L44Q	WT	–	WT	–	LVX, NA, GM, CRO, AN, GAT, F/M, FOS, CIP, FEP		AM, D, MI, C, TE
AK88-R2	ND	8	>128	0.25	A28T R114L	WT	–	WT	–	LVX, GM, CRO, AN, GAT, F/M, FOS, CIP, FEP	NA	AM, D, MI, C, TE
HK10-R1	ND	32	0.25	0.25	WT	Δ14nt (g58-t71) (frameshift)	–	WT	–	LVX, GM, CRO, AN, GAT, F/M, FOS, CIP, FEP	NA	AM, D, MI, C, TE
HK10-R2	ND	16	0.25	0.25	WT	WT	–	WT	–	LVX, GM, CRO, AN, GAT, F/M, FOS, CIP, FEP		AM, NA, D, MI, C, TE
HK10-R3	ND	16	0.25	0.25	T119S	WT	–	WT	–	LVX, GM, CRO, AN, GAT, F/M, FOS, CIP, FEP	NA	AM, D, MI, C, TE
HK2-R1	ND	4	0.25	1	Insertion of 7nt at +205 (frameshift)	WT	–	WT	–	AN, FOS		AM, CRO, F/M, NA, FEP, GM, CIP, LVX, GAT, D, C, MI, TE
**Animal isolates**												
AK267	ST15	32	128	0.25	A19V	△G139^b^ (frameshift)	+WT	Insertion of IS*5*-like between +51, +52	–	FOS, FEP, AN, CRO	GM	D, MI, CIP, LVX, NA, AM, GAT, C, F/M, TE
AK291	ST37	8	64	0.25	WT	R90G	+WT	ΔT72 (frameshift)	–	FEP, AN, CRO	FOS	D, MI, CIP, LVX, NA, AM, GAT, C, F/M, TE, GM
AK294	ST11	8	>128	0.25	△*ramR*^a^ locus	WT	–	WT	–	AN	FEP	D, MI, CIP, LVX, NA, AM, GAT, C, F/M, TE, FOS, CRO, GM
AK297	ST1326	16	0.25	0.25	WT	WT	+WT	WT	–	FEP, AN, CRO	FOS, GM	D, MI, CIP, LVX, NA, AM, GAT, C, F/M, TE
AK298	ST147	8	0.25	0.12	Y59stop	WT	–	WT	–	GM, FEP, AN, CRO		FOS, D, MI, CIP, LVX, NA, AM, GAT, C, F/M, TE
AK299	ST45	8	0.25	0.12	Q122stop	WT	+WT	WT	–	FOS, AN	FEP, C	D, MI, CIP, LVX, NA, AM, CRO, GAT, F/M, GM, TE
AK513	ST37	8	>128	0.25	WT	R90G	+WT	Insertion of IS*3*-like between +112, +113	–	FOS, FEP, AN, CRO	GM	D, MI, CIP, LVX, NA, AM, GAT, C, F/M, TE
**Human isolates**												
HK5	ST377	4	0.12	16	Insertion of 3nt at +343	WT	–	WT	OXA-48	AN	FOS	LVX, D, MI, CIP, AM, NA, GM, CRO, GAT, FEP, TE, C, F/M
HK6	ST16	4	0.12	8	I141T	△G139^b^ (frameshift)	+WT	WT	OXA-48	FOS		LVX, D, MI, CIP, AM, AN, NA, GM, CRO, GAT, FEP, F/M, TE, C
HK98	ST147	4	0.12	128	G180D	WT	–	WT	NDM-1		FOS	D, MI, LVX, CIP, AM, NA, GM, CRO, GAT, FEP, TE, AN, C, F/M
HK156	ST893	4	64	32	WT	△G139^b^ (frameshift)	+WT	Premature termination by nonsense mutation at nt88	OXA-48	AN, C	FOS, F/M	MI, D, CIP, LVX, AM, NA, GM, CRO, GAT, FEP, TE
HK157	ST2935	8	>128	64	Δ12nt (a205-c216), I141T	Δ12nt (a430-g441)	–	Premature termination by nonsense mutation at nt88	OXA-48 NDM-5	FOS	C	MI, D, CIP, LVX, AM, NA, GM, CRO, GAT, FEP, TE, AN, F/M

*WT, wild type; ND, not determined, S, susceptible; I, intermediate; R, resistant.*

*IPM, imipenem; TGC, tigecycline, Col, colistin; FOS, fosfomycin; D, doxycycline; TE, tetracycline; MI, minocycline; NA, nalidixic acid; CIP, ciprofloxacin; LVX, levofloxacin; GAT, gatifloxacin, GM, gentamicin; AN, amikacin; AM, ampicillin; CRO, ceftriaxone; FEP, cefepime; C, chloramphenicol; F/M, nitrofurantoin.*

*^*a*^Δ*ramR* locus, not amplifiable with all primers used in this study.*

*^*b*^Deletion of one of the guanines at positions 134–139.*

### *In vitro* Selection of *Klebsiella pneumoniae* Mutants With Reduced Susceptibility to Tigecycline

Three TGC-susceptible *K. pneumoniae* isolates (TGC MICs = 0.25 mg/l) were exposed to increasing concentrations of TGC. As 50% of the TGC-NSKP isolates in this study were characterized with co-resistance to colistin and TGC, we included one TGC-S but Col-R isolate among the *in vitro* selected isolates to see if resistance to colistin facilitates development of TGC resistance. Overall, it took 21–23 selection cycles to obtain six TGC-NSKP isolates with TGC MICs of 32 (*n* = 1 mutant), 16 (*n* = 3 mutants), 8 (*n* = 1 mutant), and 4 mg/l (*n* = 1 mutant) from three parental TGC-S isolates (Table 2). There was no significant difference between the selection cycles required to reach a TGC-NSKP isolate among the ColR and ColS isolates. Interestingly, all progenitor TGC-S isolates that were susceptible to tetracycline, minocycline, doxycycline, nalidixic acid, and chloramphenicol before induction became fully resistant (or showed intermediate susceptibility) to these antibiotics upon TGC non-susceptibility induction.

### *Detection and* Sequence Ana*lysis of* R*esistance* Conferring Genes

The plasmid-encoded *tetX* and *tetX1* genes were not detected in any of the TGC-NSKP isolates of both origins. All TGC-NSKP isolates of human, animal, and *in vitro* selected origins revealed wild-type RpsJ and TetA (if present). The *ramR* amplicons were obtained for 17 out of 18 TGC-NSKP isolates with the exception of isolate AK294, which did not yield any PCR product using the two pairs of primers used in this study. Eleven out of eighteen TGC-NSKP isolates (61%) (*n* = 3 animal, 4 human, and 4 *in vitro* selected mutants) revealed mutated/altered *ramR* gene. The observed RamR alterations included premature termination by nonsense mutations at amino acid positions 59 [TAC(Y) > TAA] and 122 [CAG (Q) > TAG]; a 12-nt deletion at positions 205–216; a 3-nt (AGC, serine) insertion at position +343 (between +342, +343); substitutions A19V, G180D, and I141T found among human and animal TGC-NSKP isolates and frameshift mutation due to insertion of 7 nt at position +205; and substitutions L44Q, A28T+R114L, and T119S observed among *in vitro* selected mutants. Moreover, AcrR alterations were observed among seven (38%) TGC-NSKP isolates (three animal isolates, three human isolates, and one *in vitro* selected mutant). A frameshift mutation resulting from deletion of one of the six guanines at positions 134–139 was the most common AcrR alteration identified among both animal (*n* = 1) and human isolates (*n* = 2). AcrR R90G substitution was found among two animal isolates, both of which carried a wild-type RamR protein. Also a frameshift mutation resulting from 14-nt deletions (g58-t71) was detected in one *in vitro* selected mutant (TGC MIC = 32 mg/l) carrying a wild-type RamR. One TGC-NSKP human isolate co-harbored two 12-nt deletion in *ramR* (a205-c216) and *acrR* (a430-g441) genes (Table 2). Among seven colistin-resistant isolates, five harbored inactivated/mutated MgrB due to premature termination by nonsense mutations, insertion of IS elements, and frameshift mutation as shown in Table 2. The genes encoding for carbapenemases were detected in all IPM-resistant human isolates, with three isolates harboring *bla*_OXA__–__48_, one carrying *bla*_NDM__–__1_, and one co-harboring *bla*_OXA__–__48_ and *bla*_NDM__–__5_. Two TGC-RKP isolates with TGC MIC = 16 mg/l carried wild-type RamR and AcrR proteins (Table 2).

### *Expression* Levels *of the AcrAB* Pump

**FIGURE 1:**
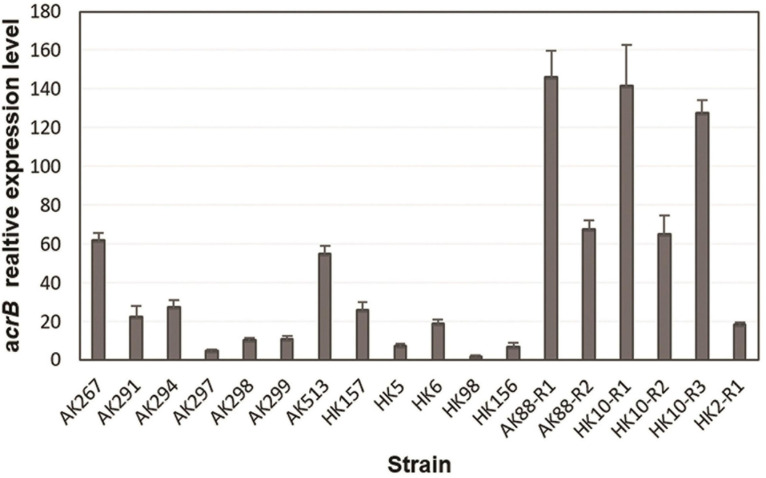
Relative expression of *acrB* gene from tigecycline (TGC)-non-susceptible isolates of *Klebsiella pneumoniae* from different origins determined by RT-qPCR.

To see if TGC non-susceptibility was linked to overexpression of AcrAB pump, the expression level of *acrB* gene was quantified by RT-qPCR analysis. A constitutively expressed housekeeping gene *rpsL* was used as a control and the TGC-susceptible clinical strain HK10 as a reference for the data analysis of animal and human isolates. For the *in vitro* selected mutants, the expression level was compared with that of their TGC-S parental strains. The *in vitro* selected TGC-NSKP isolates showed the highest expression levels (18- to 146-fold) among the studied isolates. Since the expression level of *acrB* in all three TGC-S parental isolates (K88-S, K10-S, and K2-S) were similar (representing with similar Δ_CT_), there was no significant difference when *acrB* expression of *in vitro* selected mutants was compared with that of parental TGC-S or with the TGC-S clinical isolate HK10-S. The TGC-RKP isolates of animal and human origins displayed levels of *acrB* expression that were 5- to 62-fold and 2- to 26-fold higher than those of control HK10, respectively (Figure 1). These data support the hypothesis that increased expression of *acrB* is associated with increased MICs of TGC.

## Discussion

Multidrug-resistant isolates of *K. pneumoniae* have emerged in past years as results of wide application of antibacterial agents in both human and veterinary medicine. Indeed, consumption of human antibiotics in veterinary practices has been blamed for contributing to the magnitude of current antibiotic resistance crisis. Emergence of antibiotic resistance among commensal bacteria propagated in food animals, posing a great challenge for human health since they have the potential to reach human hosts through the food chain or transmit their mobile resistance elements to human pathogens (Founou et al., 2016). Resistance of *K. pneumoniae* to various classes of antibiotics can be mediated by a variety of mechanisms, among which extrusion of antibiotics using efflux machineries such as AcrAB has been well-studied. Due to its broad substrate range property, AcrAB pump has been found to mediate resistance to a different families of antibiotics such as penicillins, cephalosporins, fluoroquinolones, macrolides, chloramphenicol, and tetracyclines (Xu et al., 2021). In the current study, screening among commensal bacteria of broiler chickens revealed six TGC-RKP isolates that were also resistant to quinolones, chloramphenicol, nitrofurantoin, and other tetracyclines. There are currently no TGC-containing products authorized for the veterinary use in Iran, and it is likely that selective pressure caused by application of other antibiotics such as older tetracyclines, florfenicol, or enrofloxacin may contribute to TGC non-susceptibility in animal commensal bacterial isolates. In a recent study from China, five TGC-RKP isolates that all belonged to ST1 were identified from the fecal samples of healthy chickens. Resistance to TGC as well as other antibiotics in these isolates was found to be mediated by a novel plasmid-mediated RND efflux pump gene cluster, designated *tmexCD1-toprJ1* (Lv et al., 2020). Li et al. also reported isolation of TGC-R *Klebsiella aerogenes* (TGC MIC = 32 mg/l) from the feces of a chicken farm, which contained the *bla*_NDM__–__9_ and *tet*(A) variant genes (Li et al., 2021). Analyzing the genetic relatedness of TGC-NSKP isolates of both origins using MLST revealed high genetic diversity among the isolates with 12 TGC-NSKP isolates belonging to 10 distinct STs, among which ST147 was found as a common type between two human and animal isolates. Most of the animal isolates belonged to STs, which are commonly reported from different human infections including ST11 (Gu et al., 2018), ST37 (Zhao et al., 2019), ST15, and ST147 (Yan et al., 2015; Chen et al., 2020). Co-resistance to TGC and colistin was observed among animal isolates belonging to ST11, ST37, and ST15. Other studies have also reported occurrence of TGC and colistin co-resistance among *K. pneumoniae* isolates belonging to ST37 and ST11 (Taniguchi et al., 2017; Xu et al., 2020). A recent study also reported detection of colistin and TGC-RKP (ST29) in municipal wastewater influents from Japan (Hayashi et al., 2021). All human TGC-NSKP isolates showed co-resistance to carbapenems and carried genes encoding for two different carbapenemases. The isolate HK157 belonging to ST2935 revealed co-resistance to three last-resort antibiotics and co-harbored a truncated *mgrB* gene, mutant *ramR* and *acrR* genes, and *bla*_OXA__–__48_ and *bla*_NDM__–__5_ carbapenemase genes. This is the first study reporting detection of *bla*_NDM__–__5_ among clinical CRKP isolates from Iran.

Analysis of the genes, AcrR (AcrAB repressor) and the RamR regulator, revealed inactivating genetic alterations in both proteins. In 83% of isolates (*n* = 15 out of 18) mutation/deletions in *ramR* (*n* = 8 isolates) or *acrR* (*n* = 4) or both (*n* = 3) was identified. The *ramR* was not detected in one isolate (AK294) using two pairs of primers used for amplification of the *ramR* gene, suggesting deletion of *ramR* locus in this isolate. In two animal isolates, RamR was truncated due to nonsense mutation, which resulted in production of 58 and 121 amino acid long proteins instead of wild-type protein with 193 amino acids. The RamR Q122 stop substitution has been previously reported among TGC-NSKP isolates by several other studies presenting this alteration as a common resistance mechanism among *K. pneumoniae* isolates (He et al., 2015; Chiu et al., 2017b; Li et al., 2017; Park et al., 2020). In addition to two previously reported RamR A19V and L44Q mutations (reported as L44R) (Chiu et al., 2017b), five novel amino acid substitutions, including I141T, G180D, A28T, R114L, and T119S, were identified, with the latter three changes being detected after resistance induction in *in vitro* selected mutants. The A19V substitution, which was also predicted by the PROVEAN tool to be a neutral change, has been previously demonstrated to have no effect on the TGC MICs (Chiu et al., 2017b). While RamR A28T, R114L, and G180D substitutions were predicted by the PROVEAN tool to have a deleterious impact on protein structure (PEOVEAN scores –3.6, –4, and –6.8, respectively), the I141T and T119S substitutions (with PEOVEAN scores 0.21 and –2.44 (very close to prediction cutoff = –2.5), respectively) were predicted to be neutral changes. An isolate-carrying RamR I141T substitution co-harbored an AcrR inactivating mutation (frameshift), indicating that AcrR alteration might be the main mediator of TGC non-susceptibility in this bacterium. However, the exact role of these novel mutations in elevation of TGC MICs requires further studies by confirmatory assays. In one *in vitro* selected mutant and a clinical TGC-R isolate nucleotide insertion (7nt) and deletion (12 nt) at position +205 was observed, respectively, indicating that this position is more prone to alterations upon resistance development. Among the 18 TGC-NSKP, mutations/deletions within AcrR were detected among seven isolates from which six carried a wild-type RamR or RamR substitutions, which were predicted or previously demonstrated to be neutral changes. The novel AcrR R90G substitution detected in two TGC-R animal isolates belonging to ST37 was predicted by the PROVEAN tool to have a deleterious effect on the functionality of the protein (PROVEAN score = –5.9). The AcrR frameshift mutation mediated by deletion of one of the guanines at positions 134–139 was detected in three TGC-NSKP isolates of both origins that belonged to different STs representing this region as a mutation-prone position within *acrR* gene. In two TGC-RKP isolates with overexpressed AcrB (AK297 and HK10-R2, TGC MIC = 16 mg/l), no alteration was identified in any of the studied genes, indicating that alterations in promoter region of the studied genes or other loci (*marR* or *soxR*; Bratu et al., 2009; Veleba and Schneiders, 2012) are probably involved in resistance development in these isolates. Gene expression analysis revealed an association between overexpression of AcrAB efflux pump and TGC non-susceptibility. However, there was no correlation between the TGC MICs and the level of *acrB* expression, as some isolates with similar MICs exhibited different expression levels. This suggests the possibility of contribution of other resistance mechanisms to increased TGC MICs.

## Conclusion

A combination of genetic alterations in AcrR and RamR mediated the overexpression of AcrAB efflux pump and subsequently TGC non-susceptibility among the majority of human, animal, and *in vitro* selected mutants studied in this work. The most worrisome finding in our study was detection of multidrug-resistant isolates with co-resistance to two last-resort human antibiotics (TGC and colistin) belonging to sequence types commonly implicated in human infections (ST11, ST37, and ST15) among commensal bacteria of food animals. This can be considered a great threat to human health due to probability of transmission of these bacteria to humans through the food chain or direct contact. Acquiring carbapenem resistance among these isolates would be the most troublesome event. While the TGC exposure history among the human isolates was not clear, emergence of TGC resistance among animal isolates (which are not exposed to TGC) is an issue of great concern. It is speculated that selective pressure caused by other antimicrobials in particular tetracycline families (as one of the most used antimicrobials in food animals) may have contributed to increased TGC MICs in these isolates probably through the overexpression of AcrAB efflux pump. Therefore, immediate actions need to be taken to restrict/minimize the use of human antibiotics (at least not as growth promoters) in food animals to prevent the emergence and dissemination of antibiotic-resistant bacteria through the food chain.

## Data Availability Statement

Publicly available datasets were analyzed in this study. This data can be found here: GenBank; accession numbers MW653710-MW653741.

## Author Contributions

MM performed the experiments, analyzed the experiment data, and drafted the manuscript. MH and HM designed the experiments, analyzed the experiment data, and wrote the manuscript. All authors read and approved the final manuscript.

## Conflict of Interest

The authors declare that the research was conducted in the absence of any commercial or financial relationships that could be construed as a potential conflict of interest.

## Publisher’s Note

All claims expressed in this article are solely those of the authors and do not necessarily represent those of their affiliated organizations, or those of the publisher, the editors and the reviewers. Any product that may be evaluated in this article, or claim that may be made by its manufacturer, is not guaranteed or endorsed by the publisher.

## Footnotes

1
http://bigsdb.pasteur.fr/klebsiella/klebsiella.html

2
https://isfinder.biotoul.fr
